# Dating the *Cryptococcus gattii* Dispersal to the North American Pacific Northwest

**DOI:** 10.1128/mSphere.00499-17

**Published:** 2018-01-17

**Authors:** Chandler C. Roe, Jolene Bowers, Hanna Oltean, Emilio DeBess, Philippe J. Dufresne, Scott McBurney, David P. Overy, Bodo Wanke, Colleen Lysen, Tom Chiller, Wieland Meyer, George R. Thompson, Shawn R. Lockhart, Crystal M. Hepp, David M. Engelthaler

**Affiliations:** a Translational Genomics Research Institute, Flagstaff, Arizona, USA; b Washington State Department of Health, Shoreline, Washington, USA; c Public Health Division, Oregon Health Authority, Salem, Oregon, USA; d Laboratoire de Santé Publique du Québec, Institut National de Santé Publique du Québec, Charlottetown, Prince Edward Island, Canada; e Canadian Wildlife Health Cooperative, Atlantic Veterinary College, University of Prince Edward Island, Charlottetown, Prince Edward Island, Canada; f Agriculture and Agrifood Canada, Ottawa Research and Development Centre, Ottawa, Ontario, Canada; g National Institute of Infectious Diseases Evandro Chagas, FIOCRUZ, Rio de Janeiro, Brazil; h Mycotic Disease Branch, Centers for Disease Control and Prevention, Atlanta, Georgia, USA; i Molecular Mycology Research Laboratory, Center for Infectious Diseases and Microbiology, Marie Bashir Institute for Emerging Infections and Biosecurity, Westmead Clinical School, Sydney Medical School, The University of Sydney, Westmead, New South Wales, Australia; j Westmead Hospital, Westmead Institute for Medical Research, Westmead, New South Wales, Australia; k University of California, Davis, Davis, California, USA; l School of Informatics, Computing, and Cyber Systems, Northern Arizona University, Flagstaff, Arizona, USA; Duke University Medical Center

**Keywords:** *Cryptococcus*, genomics, molecular epidemiology, mycology

## Abstract

The emergence of *Cryptococcus gattii*, previously regarded as a predominantly tropical pathogen, in the temperate climate of the North American Pacific Northwest (PNW) in 1999 prompted several questions. The most prevalent among these was the timing of the introduction of this pathogen to this novel environment. Here, we infer tip-dated timing estimates for the three clonal *C. gattii* populations observed in the PNW, VGIIa, VGIIb, and VGIIc, based on whole-genome sequencing of 134 *C. gattii* isolates and using Bayesian evolutionary analysis by sampling trees (BEAST). We estimated the nucleotide substitution rate for each lineage (1.59 × 10^−8^, 1.59 × 10^−8^, and 2.70 × 10^−8^, respectively) to be an order of magnitude higher than common neutral fungal mutation rates (2.0 × 10^−9^), indicating a microevolutionary rate (e.g., successive clonal generations in a laboratory) in comparison to a species’ slower, macroevolutionary rate (e.g., when using fossil records). The clonal nature of the PNW *C. gattii* emergence over a narrow number of years would therefore possibly explain our higher mutation rates. Our results suggest that the mean time to most recent common ancestor for all three sublineages occurred within the last 60 to 100 years. While the cause of *C. gattii* dispersal to the PNW is still unclear, our research estimates that the arrival is neither ancient nor very recent (i.e., <25 years ago), making a strong case for an anthropogenic introduction.

**IMPORTANCE** The recent emergence of the pathogenic fungus *Cryptococcus gattii* in the Pacific Northwest (PNW) resulted in numerous investigations into the epidemiological and enzootic impacts, as well as multiple genomic explorations of the three primary molecular subtypes of the fungus that were discovered. These studies lead to the general conclusion that the subtypes identified likely emerged out of Brazil. Here, we conducted genomic dating analyses to determine the ages of the various lineages seen in the PNW and propose hypothetical causes for the dispersal events. Bayesian evolutionary analysis strongly suggests that these independent fungal populations in the PNW are all 60 to 100 years old, providing a timing that is subsequent to the opening of the Panama Canal, which allowed for more direct shipping between Brazil and the western North American coastline, a possible driving event for these fungal translocation events.

## Abstract (precis)

The recent emergence of the pathogenic fungus *Cryptococcus gattii* in the Pacific Northwest (PNW) resulted in numerous investigations into the epidemiological and enzootic impacts, as well as multiple genomic explorations of the three primary molecular subtypes of the fungus that were discovered. These studies lead to the general conclusion that the subtypes identified likely emerged out of Brazil. Here, we conducted genomic dating analyses to determine the ages of the various lineages seen in the PNW and propose hypothetical causes for the dispersal events. Bayesian evolutionary analysis strongly suggests that these independent fungal populations in the PNW are all 60 to 100 years old, providing a timing that is subsequent to the opening of the Panama Canal, which allowed for more direct shipping between Brazil and the western North American coastline, a possible driving event for these fungal translocation events.

## INTRODUCTION

Combining epidemiology with microbial evolution analyses in a historical context is critical to understanding the nature of newly occurring infectious diseases. Diseases may emerge in a new region due to recent pathogen translocation events (e.g., West Nile virus in the United States in 1999 [1], Ebola virus in western Africa in 2014 [2], and Zika virus in Brazil in 2016 [3]). Many times “emerging diseases” are only emerging in our understanding of previously undetected endemic disease (e.g., *Legionella* in Philadelphia in 1976 [4] and hantavirus in the American Southwest in 1993 [5]). The appearance of *Cryptococcus gattii* in the Pacific Northwest (PNW) may represent both of these models (note that while the species nomenclature of *C. gattii* is currently under debate [6, 7], for consistency with previous and ongoing studies, we use the traditional *C. gattii* nomenclature herein, with a focus on the major molecular type VGII).

The source and timing of the emergence of *C. gattii* in the PNW have been a challenge to public health and mycology researchers since cryptococcosis seemingly first appeared in British Columbia in 1999 (8, 9). Early studies elucidated the highly clonal nature of the newly identified subtypes of the VGII major molecular type—VGIIa, VGIIb, and eventually, VGIIc—in the Oregon-Washington region (9, 10). More recent studies have identified the origin of *C. gattii*, including these subtypes, to be South America, likely Brazil (11–13), where *C. gattii* is endemic. However, their apparently sudden appearance, with novel phenotypes and relatively widespread nature, across the North American PNW remained a genomic enigma (12).

Here, we apply Bayesian analysis-based genomic dating to understand the timing of the PNW emergence(s) in order to better elucidate the causes of the *C. gattii* translocation events and dispersal to and within North America. In an effort to provide estimates of time to most recent common ancestor (TMRCA), we implemented an established dating method and combined that with public health surveillance and epidemiology, along with a historical understanding of global anthropogenic events, to establish a hypothesis of “how and when” for the dispersal of *C. gattii*.

## RESULTS

**FIG 1:**
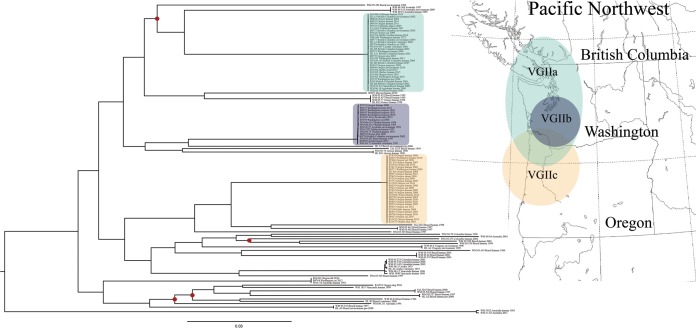
Maximum-likelihood phylogeny and geographic locations of the PNW *Cryptococcus gattii* VGII lineages. This tree is based on 289,240 total SNPs using the TVM+ASC+G4 nucleotide substitution model and includes 134 genomes that collectively cover 87.57% of the R265 BC human 2001 reference genome. Within this lineage, three clonal subclades exist, VGIIa, VGIIb, and VGIIc. This tree includes 1,000 bootstrap pseudoreplicates; nodes with bootstrap values less than 95% are denoted by red circles. Geographic representations of the three PNW clones are a generalized depiction of the primary impact region of each in the PNW.

A total of 134 *Cryptococcus gattii* whole genomes, 112 of which were previously published, representing both the global VGII lineages and the Pacific Northwest subtypes, were included in the maximum-likelihood tree (Fig. 1). This tree is based on 289,240 total single-nucleotide polymorphisms (SNPs) with 213,653 informative SNPs, using the TVM+ASC+G4 nucleotide substitution model (14). This SNP matrix had a quality breadth of coverage of 87.57% of the reference genome R265.

The *C. gattii* subtypes in the PNW are highly clonal, with 412, 153, and 268 SNPs within VGIIa, VGIIb, and VGIIc, respectively (see Fig. S1 to S3 in the supplemental material), which is consistent with preliminary findings (9, 10) but unlike other *C. gattii* populations in the United States (15). The newly sequenced 2015/2016 isolates of VGIIa, VGIIb, and VGIIc all grouped within the PNW clades. Two 2015 autochthonous VGIIa isolates from Québec, Canada (16), that grouped together within the PNW clade, seemingly derived from an Oregon strain lineage, and one 2015 wildlife isolate from Nova Scotia (17) that grouped within the primary VGIIb PNW clade seemingly derived from a Washington lineage. The three PNW genotypes all continue to be clonal, with no evidence of recombination based on the pairwise homoplasy index (PHI) statistic.

10.1128/mSphere.00499-17.1 FIG S1  Maximum-likelihood phylogeny of *C. gattii* VGIIa data set. The VGIIa tree is based on 412 SNPs using the K2P+ASC nucleotide substitution model and includes 29 genomes that collectively cover 96.9% of the R265 BC human 2001 reference genome. The PHI test did not find statistically significant evidence for recombination in the VGIIa SNP matrix (*P* = 1.0). Download FIG S1, JPG file, 1.1 MB. Copyright © 2018 Roe et al. 2018 Roe et al. This content is distributed under the terms of the Creative Commons Attribution 4.0 International license.

10.1128/mSphere.00499-17.2 FIG S2  Maximum-likelihood phylogeny of *C. gattii* VGIIb data set. The VGIIb tree is based on 153 SNPs using the K2P+ASC nucleotide substitution model and includes 8 genomes with a quality breadth of coverage of the B11567 Nova Scotia deer VGIIb 2015 reference genome of 97.1%. The PHI test statistic, *P* = 1.0, indicates no significant evidence for recombination in the VGIIb SNP matrix. Download FIG S2, JPG file, 0.6 MB. Copyright © 2018 Roe et al. 2018 Roe et al. This content is distributed under the terms of the Creative Commons Attribution 4.0 International license.

10.1128/mSphere.00499-17.3 FIG S3  Maximum-likelihood phylogeny of *C. gattii* VGIIc data set. The VGIIc tree included 268 SNPs using the K2P+ASC nucleotide substitution model. Here, 29 genomes had a quality breadth of coverage of the B11468 OR human 2016 reference genome of 96.4%. The PHI test again yielded *P* = 1.0. Download FIG S3, JPG file, 1.7 MB. Copyright © 2018 Roe et al. 2018 Roe et al. This content is distributed under the terms of the Creative Commons Attribution 4.0 International license.

The root-to-tip regressions identified various degrees of clocklike behavior among the three genotypes, with *R*^2^ values of 0.5971, 0.661, and 0.0745, suggesting that VGIIa and VGIIb have strong clocklike behavior while VGIIc has weak clocklike behavior (Fig. S4 to S6). However, because all three genotypes have positive regression slopes, molecular clock analyses are appropriate and reliable for mutation rate estimation (18). The best-fitting model from MEGA7 that was also available in the BEAST (Bayesian evolutionary analysis by sampling trees**)** software was implemented: the HKY model was applied to both the VGIIa and VGIIb data sets, while the best-fitting model for the VGIIc data set was the TN93 model.

10.1128/mSphere.00499-17.4 FIG S4  Root-to-tip analyses to assess the temporal signal (the clocklike behavior) in the VGIIa data set of point mutation accumulation over time. The VGIIa SNPs illustrate strong clocklike behavior with an *R*^2^ of 0.5971. The 10,000-date randomization permutation distribution is included, showing a *P* value of 0.076, suggesting our regression analysis is on the cusp of being better than we would observe from random chance. Download FIG S4, JPG file, 0.2 MB. Copyright © 2018 Roe et al. 2018 Roe et al. This content is distributed under the terms of the Creative Commons Attribution 4.0 International license.

10.1128/mSphere.00499-17.5 FIG S5  Root-to-tip analysis of the VGIIb group resulting in an *R*^2^ value of 0.661 and a positive slope, suggesting strong clocklike behavior. The 10,000-date randomization permutation distribution for the VGIIb data set had a *P* value of 0.268. Our observed *R*^2^ value is only better than 73% of what we observe from random chance. Download FIG S5, JPG file, 0.2 MB. Copyright © 2018 Roe et al. 2018 Roe et al. This content is distributed under the terms of the Creative Commons Attribution 4.0 International license.

10.1128/mSphere.00499-17.6 FIG S6  The VGIIc regression plot is diffuse, with an *R*^2^ value of 0.074, suggesting weak clocklike behavior. The 10,000-date randomization permutation distribution for VGIIc produced a *P* value of 0.294. Our observed *R*^2^ value is only better than 70% of what we observe from random chance. Download FIG S6, JPG file, 0.2 MB. Copyright © 2018 Roe et al. 2018 Roe et al. This content is distributed under the terms of the Creative Commons Attribution 4.0 International license.

The 10,000-date randomization permutation testing on the VGIIa sample set produced a *P* value of 0.076, suggesting that the *R*^2^ value produced in the regression analysis was better than 9,240 date randomized regression analyses and that our results are significantly different from what would be expected from random chance (Fig. S4). The date randomization testing for VGIIb and VGIIc produced *P* values of 0.268 and 0.294, respectively, showing that our observed *R*^2^ values were not statistically different than random chance (Fig. S5 and S6).

The estimated mutation rates for VGIIa and VGIIc are extremely similar by BEAST calculations; the VGIIa mutation rate was calculated to be 1.59 × 10^−8^ SNPs per base per year (95% highest posterior density [HPD], 5.54 × 10^−9^ to 2.93 × 10^−8^), while that of VGIIc is 1.59 × 10^−8^ SNPs per base per year (95% HPD, 5.54 × 10^−9^ to 2.04 × 10^−8^). Even though VGIIa’s root-to-tip regression was clocklike compared to that of VGIIc, both genotypes exhibit low rates of evolutionary change. The estimated mutation rate for VGIIb was calculated to be nearly twice as high as those of the VGIIa and VGIIc lineages, with 2.70 × 10^−8^ SNPs per base per year (95% HPD, 6.75 × 10^−9^ to 5.23 × 10^−8^).

**FIG 2:**
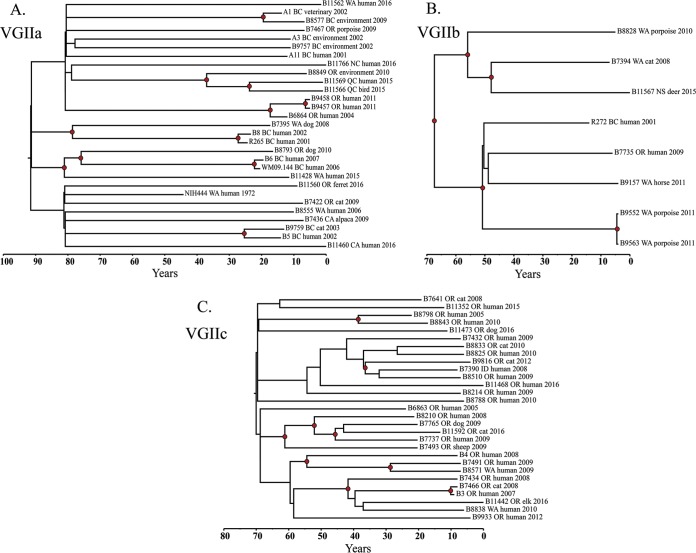
Bayesian phylogenetic analyses of *C. gattii* samples. BEAST 1.8.4 was used to produce calibrated phylogenies with the mean TMRCA estimates, which were 87.99 years ago for the VGIIa data set (A), 81.43 years ago for the VGIIb data set (B), and 66.29 years ago for the VGIIc data set (C). The tips of the branches correspond to the year of sampling. Red nodes represent internal nodes with posterior probability support of >0.95.

The time to most recent ancestor (TMRCA) was calculated for each sample set from the BEAST analysis; the mean TMRCA for the PNW VGIIa sample set was estimated at 87.99 years ago (95% HPD, 53.87 to 173.76), the mean TMRCA for the PNW VGIIb sample set was estimated at 81.43 years ago (95% HPD, 27.93, 187.95), and the mean TMRCA for the VGIIc sample set was estimated at 66.29 years ago (95% HPD, 26.91, 115.43) (Fig. 2).

## DISCUSSION

Between 2005 and 2013, a total of 273 *Cryptococcus gattii* cases from both human and veterinary sources were reported in the PNW (19), with new cases still reported today. Understanding the emergence and continual evolution of this pathogen in a novel environment is critical to the understanding of the ongoing epidemiology of cryptococcal disease in this region and may be important to studying the evolution of other emerging pathogens. This unexpected and ongoing emergence of what was previously thought to be predominantly a tropical pathogen spurred several questions regarding the genetic and geographic origin of this fungus (12, 20) and, pointedly, the timing of the introduction of *C. gattii* into the PNW. Here, we applied a Bayesian approach to narrow in on a dispersal estimate.

Previous studies estimating the divergence time of *C. neoformans* and *C. gattii* applied a common neutral mutation rate of 2.0 × 10^−9^. We estimated the mean substitution rates for all three genotypes to be between 1.59 × 10^−8^ and 2.70 × 10^−8^, an order of magnitude higher than previously described rates (21). Several studies have shown microevolutionary rates (e.g., successive clonal generations in a laboratory) to be consistently higher than a species’ macroevolutionary rates (e.g., when using fossil records), which are likely affected by purifying selection that becomes evident over longer periods of time (22–27). The latter likely includes other nonclonal reproduction effects that would not impact the likely asexual life history of *C. gattii* in the PNW, where only one mating type has been identified (9). The clonal nature of the PNW emergences over a narrow number of years would explain the higher mutation rates.

Our results suggest that *C. gattii* has been in the PNW longer than previously hypothesized. The clinical emergence of specific major molecular types in 1999 (VGIIa and VGIIb) and 2005 (VGIIc) was likely an emergence in our understanding of previously arrived and dispersed fungi from Brazil. The polytomy effect of individual lineages dating back to the MRCA (i.e., founder populations) in each of the VGIIa, VGIIb, and VGIIc clades suggests an initial evolutionary bottleneck followed by an early intra-PNW dispersal event, and the subclades within these lineages demonstrate ongoing divergence events during their evolutionary histories on the continent. Unlike VGIIa and VGIIc, VGIIb was reported to have been introduced to the PNW on at least two occasions (12); in this analysis, we only included the members of the primary (i.e., major) PNW clade, which now includes the 2015 Nova Scotia isolate, as the other, minor clade contained only two isolate genomes with no new members identified since the original analysis (12). The increased attention following the initial case cluster of VGIIa on Vancouver Island in 1999 likely led to an increase of case findings and the subsequent finding of additional “novel” subtypes elsewhere in the region (e.g., VGIIc in Oregon) (9). Our common centurial dating for the seemingly independent emergences of the PNW *C. gattii* subtypes suggests a possible common mechanism for their translocation. A search for causes of transmission can now focus on events and means of transmission that occurred at the time of or shortly preceding the MRCA dates.

### The Teddy Roosevelt effect.

**FIG. 3:**
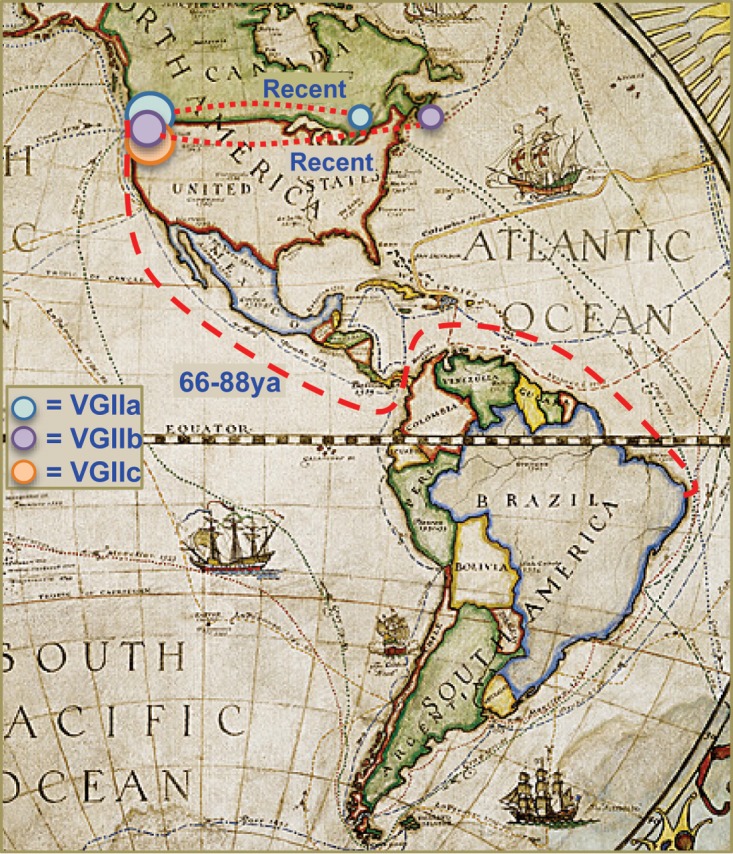
Geographic representation of the timing of dispersal of the VGII lineages to the PNW. ya, years ago. (Adapted from reference 60 with permission from National Geographic.)

Given the commonality of just under 100 years for the PNW arrival of the VGIIa, -b, and -c subtypes and given their lack of known presence elsewhere on the North American continent (with three notable exceptions: a single Florida VGIIb isolate [28], the recently discovered Québec cluster of VGIIa [16, 29], which seems to have derived from the PNW VGIIa population, and the single VGIIb isolate from a deer in Nova Scotia [17], likely derived from the PNW VGIIb population), it is logical to hypothesize that these *C. gattii* subtypes arrived in the PNW region shortly into the 20th century. It is possible that populations arrived elsewhere on the continent and failed to become established; a similar scenario was seen with the concurrent spread of *Yersinia pestis* by boats from Asia to multiple U.S. ports around the turn of the 20th century that only became enzootic through the ports in California (30). What appears to be clear is that there was an outward spread of the PNW *C. gattii* populations from the PNW coastal regions to locales further inland (including across the country of Canada). These findings would suggest at a minimum that a translocation event or events occurred between Brazil and the PNW coast approximately 100 years ago. As shipping was minimal between Brazil and Pacific Coastal countries in South America until the opening of the Panama Canal, it follows that this historical event may have allowed for ships to carry cargo, and microbial stowaways, between these distant locations for the first time (Fig. 3).

The Panamanian isthmus acts as a land bridge between the American continents and as a natural barrier between the Pacific and Atlantic Ocean. The isthmus has played a crucial role in the geology, hydrology, biology, and climate in the region since the uprising of this intercontinental land bridge nearly 3 million years ago (31). The isthmus has also played a key role in the dispersal of microbes: notably, the fungal pathogen *Coccidioides posadasii* likely was translocated from North America to South America across the isthmus via mass movements of mammals during the later Great American Biotic Interchange events that occurred nearly a million years ago (32). After the final rising of the isthmus, a permanent land barrier was formed between the continents, separating the oceans, until just over 100 years ago. In 1914, after years of human toil, the Panama Canal, commissioned by Theodore Roosevelt, was completed. This allowed, for the first time in nearly 3 million years, water movement between the Atlantic and Pacific Oceans, subsequently allowing for cargo ships to move between the east and west coasts of the Western Hemisphere continents without having to go through dangerous waters at the southern point of South America. After the opening of the Panama Canal, shipping between ports of Brazil and western North America began in earnest.

Common goods transported out of Brazil at this time were Brazilian hardwood lumber, minerals, coffee, and rubber. While *C. gattii* is known to have been transported on (33) and to thrive on (34, 35) live trees and seeds, no contamination of lumber products has been documented previously. While we may be unable to ascertain a specific shipping product that may have carried the fungus from Brazil to the PNW and elsewhere, one hypothesis we propose is contaminated ballast water. Ballast water contamination is a well-understood problem in large vessels, and ballast has been known to contain and transport nonnative animals, algae, and microbes around the world (36). At a vessel’s source port, seawater is taken on by ships and stored in their ballast to provide vessel stability. This ballast water is released *en route* or at the destination port to correct for fluctuations in cargo weight (37). *Cryptococcus* has been shown to survive *in vitro* in saline (8), has been isolated from seawater (38, 39), and is known to cause significant morbidity and mortality in marine mammals (40–42), particularly in the PNW (43–45). Again, while only hypothetical, it is possible that a limited number of ships with contaminated ballast water could have transported these pathogens unknowingly to PNW ports and beyond. It is notable that the dominant PNW clade of VGIIb also shares a very recent common ancestor with multiple lineages in Southeast Asia. Again, a shipping vessel from Brazil carrying goods (and contaminants) to the PNW could also have caused the translocation to Asia at this time, given that the new transcontinental seaway would have allowed shipping to these markets as well.

An alternate and intriguing hypothesis for dispersal to Malaysia and elsewhere in Southeast Asia is the possible contamination of rubber tree (*Hevea brasiliensis*) seeds, which were infamously “stolen” and exported to Britain by Henry Wickham in 1876 to establish British-controlled rubber tree plantations in Ceylon (now Sri Lanka) and Malay (modern day Malaysia and Singapore) and, subsequently, to British colonies in Africa and India (46). While not previously shown to be associated with *Hevea* seeds, *C. gattii* contamination of other tree seeds, such as *Eucalyptus* spp., has been well documented (33). Brazil, attempting to maintain global control of the rubber trade, tried to prevent the shipment of such seeds, limiting other possible such exports out of Brazil; however, the Wickham seed transport likely resulted in the crash of Brazilian rubber beginning in 1910 (46). The 1876 seed transport timeline and global movement of *Hevea* seeds at this time could represent an indirect dispersal mechanism. Along these lines, the Brazil nut (*Bertholletia excelsa*) has been an additional plant-based export out of Brazil around the world for multiple centuries; however, intense exportations began following the Brazilian rubber crash in 1910 (47), providing an appropriate temporal connection to the *C. gattii* dispersal event. Extensive analyses of well-known ongoing fungal contamination of Brazil nuts during collection and processing have not identified the presence of *Cryptococcus* spp. (47), and there is no obvious route to localized environmental contamination in the PNW from these products.

While the prevalence of recombination along with the extent of temporal signal within a sample set plays a large role in estimating genome-wide evolutionary rates (18), these sample sets show no statistical evidence of recombination to hinder these analyses. However, the date randomization permutation testing showed there was little true temporal signal within the VGIIb and VGIIc sample sets. Given the narrow sampling dates and the limited number of samples in each data set, it is possible we are only capturing a glimpse of the temporal signal and, with a wider sampling time and more samples, it would be possible to narrow our MRCA estimates.

Whatever the cause of *C. gattii* dispersal to the PNW, it is clear that those populations are neither ancient nor very recent (i.e., <25 years ago) arrivals to the region. Dispersal in the last 100 years would strongly suggest anthropogenic causes, directly or indirectly, rather than movement by natural animal migrations, as suggested with *Cryptococcus* populations (48) and other environmental pathogenic fungi (32). The identification of VGIIa in a pet store bird (sample number B11566) and a separate, unrelated case in pet store worker (sample number B11569), both in Québec, are unexpected occurrences (29), and it is of interest to note that the bird reportedly originated from British Columbia and the infecting *C. gattii* strain clearly evolved from the PNW VGIIa population, suggesting a linkage to human transport of animals as the cause. Conversely, the appearance of a PNW-derived strain of VGIIb (sample number B11567) in a Nova Scotia yearling white-tailed deer (*Odocoileus virginianus*) (17) is less explainable. There is no other current evidence of long-distance dispersal of VGIIb across the North American continent, nor is there evidence of transport of animals from the PNW to the region, as the infected deer was part of a local deer population. White-tailed deer are nonmigratory, providing further evidence of the presence of VGIIb in Nova Scotia, and this is suggestive of a unique introduction/dispersal event in the region (17). The province of Nova Scotia is a peninsula that is surrounded by four major bodies of water, and the city of Halifax, throughout its history, has been an active sea port, one of the major international points of entry for shipping into Atlantic, Canada. More investigation of the local environment is necessary to understand the history of this incident and provide estimates of regional endemicity. As the North American populations of *C. gattii* continue to evolve and disperse, it will be useful to continually apply genomic dating to understand the nature of these events and the expanding impact of these fungi on human and veterinary health.

## MATERIALS AND METHODS

### Samples.

A total of 66 genomes were included in the analysis of the three clonal lineages found in the PNW. The 22 new genomes from this study include 13 *C. gattii* recent isolates collected either in 2015 or 2016 from Washington, Oregon, and Canada, which were comprised of 7 VGIIa, 1 VGIIb, and 5 VGIIc isolates (Table 1). Previously published isolate genomes (12) were included for each genotype, including 22 VGIIa, 16 VGIIb, and 24 VGIIc genomes (Table 1). The VGII lineage tree was based off of 134 genomes, 112 of which were previously published (12), including the above-mentioned samples (Table 1). The recently described *msh2* variant lineage of VGIIa (49) was not included in the genotype-specific genomic dating analyses (described below) due to possible confounding from the hypermutation phenotype, except for the NIH444 genome, which had no identifiable impacts on the total number of SNP mutations (12).

**TABLE 1:** *C. gattii* VGII sample genomes used in this study, including 22 newly sequenced and 112 previously published genomes

Sample	Genotype	Location	Source	Yr of isolation	Accession no.	
					BioProject^a^	BioSample
R265	VGIIa	Canada	Human	2001	PRJNA244927	SAMN02851006
B6864	VGIIa	Oregon	Human	2004	PRJNA244927	SAMN02850991
B7422	VGIIa	Oregon	Cat	2009	PRJNA244927	SAMN02850993
B7436	VGIIa	California	Alpaca	2009	PRJNA244927	SAMN02850994
B8555	VGIIa	Washington	Human	2006	PRJNA244927	SAMN02850996
B8793	VGIIa	Oregon	Dog	2010	PRJNA244927	SAMN02850998
CA 1014	VGIIa	California	Human	NA^b^	PRJNA244927	SAMN02851004
ICB 107	VGIIa	Brazil	Human	1981	PRJNA244927	SAMN02851017
NIH 444	VGIIa	Washington	Human	1972	PRJNA244927	SAMN02851012
B7395	VGIIa	Washington	Dog	2008	PRJNA244927	SAMN02850992
B7467	VGIIa	Oregon	Porpoise	2009	PRJNA244927	SAMN02850995
B8577	VGIIa	Canada	Environmental	2009	PRJNA244927	SAMN02850997
B8849	VGIIa	Oregon	Environmental	2010	PRJNA244927	SAMN02850999
B11428	VGIIa	Washington	Human	2015	PRJNA388113	SAMN07738523
B11460	VGIIa	California	Human	2016	PRJNA388113	SAMN07738524
B11560	VGIIa	Oregon	Ferret	2016	PRJNA388113	SAMN07738525
B11562	VGIIa	Washington	Human	2016	PRJNA388113	SAMN07738526
B11566	VGIIa	Québec	Bird	2015	PRJNA388113	SAMN07738527
B11569	VGIIa	Québec	Human	2015	PRJNA388113	SAMN07738528
B9457	VGIIa	Oregon	Human	2011	PRJNA244927	SAMN02851000
B9458	VGIIa	Oregon	Human	2011	PRJNA244927	SAMN02851001
B9757	VGIIa	Canada	Environmental	2002	PRJNA244927	SAMN02851002
B9759	VGIIa	Canada	Cat	2003	PRJNA244927	SAMN02851003
WM 03.697	VGIIa	Canada	Veterinary	2001	PRJNA244927	SAMN02851013
WM 05.432	VGIIa	Japan/Brazil	Human	2000	PRJNA244927	SAMN02851014
WM 05.554	VGIIa	Brazil	Human	2002	PRJNA244927	SAMN02851015
WM 06.10	VGIIa	Argentina	Human	2000	PRJNA244927	SAMN02851016
WM 09.144	VGIIa	Canada	Human	2006	PRJNA388113	SAMN07738529
HL A11	VGIIa	Canada	Human	2001	PRJNA244927	SAMN02851009
HL A1	VGIIa	Canada	Veterinary	2002	PRJNA244927	SAMN02851007
HL A3	VGIIa	Canada	Environmental	2002	PRJNA244927	SAMN02851008
HL B5	VGIIa	Canada	Human	2002	PRJNA244927	SAMN02851010
HL B6	VGIIa	Canada	Human	2007	PRJNA244927	SAMN02851011
HL B8	VGIIa	Canada	Human	2002	PRJNA388113	SAMN07738530
B7735	VGIIb	Oregon	Human	2009	PRJNA244927	SAMN02851019
B8554	VGIIb	Oregon	Dog	2008	PRJNA244927	SAMN02851020
B8828	VGIIb	Washington	Porpoise	2010	PRJNA244927	SAMN02851021
B11567	VGIIb	NS, Canada^c^	Deer	2015	PRJNA388113	SAMN07738531
B7394	VGIIb	Washington	Cat	2008	PRJNA244927	SAMN02851018
B7735	VGIIb	Oregon	Human	2009	PRJNA244927	SAMN02851019
B9157	VGIIb	Washington	Horse	2011	PRJNA244927	SAMN02851022
B9552	VGIIb	Washington	Porpoise	2011	PRJNA244927	SAMN02851023
B9563	VGIIb	Washington	Porpoise	2011	PRJNA244927	SAMN02851024
B9588	VGIIb	Florida	Human	2012	PRJNA244927	SAMN02851025
B9758	VGIIb	Canada	Environmental	2002	PRJNA244927	SAMN02851026
WM 03.27	VGIIb	Australia	Environmental	1992	PRJNA244927	SAMN02851029
WM 04.71	VGIIb	Australia	Veterinary	1991	PRJNA244927	SAMN02851030
WM 04.75	VGIIb	Thailand	Human	1993	PRJNA244927	SAMN02851034
WM 05.465	VGIIb	Brazil	Human	1997	PRJNA244927	SAMN02851031
WM 06.634	VGIIb	Thailand	Human	1994	PRJNA244927	SAMN02851032
WM 06.636	VGIIb	Thailand	Human	1995	PRJNA244927	SAMN02851033
WM 2552	VGIIb	Malaysia	Human	1997	PRJNA244927	SAMN02851028
B7434	VGIIc	Oregon	Human	2008	PRJNA244927	SAMN02851037
B7491	VGIIc	Oregon	Human	2009	PRJNA244927	SAMN02851039
B7493	VGIIc	Oregon	Sheep	2009	PRJNA244927	SAMN02851040
B7641	VGIIc	Oregon	Cat	2008	PRJNA244927	SAMN02851041
B7765	VGIIc	Oregon	Dog	2009	PRJNA244927	SAMN02851043
B8210	VGIIc	Oregon	Human	2008	PRJNA244927	SAMN02851044
B8214	VGIIc	Oregon	Human	2009	PRJNA244927	SAMN02851045
B8510	VGIIc	Oregon	Human	2009	PRJNA244927	SAMN02851046
B8788	VGIIc	Oregon	Human	2010	PRJNA244927	SAMN02851048
B8798	VGIIc	Oregon	Human	2005	PRJNA244927	SAMN02851049
B8833	VGIIc	Oregon	Cat	2010	PRJNA244927	SAMN02851051
B7390	VGIIc	Idaho	Human	2008	PRJNA244927	SAMN02851059
B7432	VGIIc	Oregon	Human	2009	PRJNA244927	SAMN02851036
B6863	VGIIc	Oregon	Human	2005	PRJNA244927	SAMN02851035
B7466	VGIIc	Oregon	Cat	2008	PRJNA244927	SAMN02851038
B7737	VGIIc	Oregon	Human	2009	PRJNA244927	SAMN02851042
B8571	VGIIc	Washington	Human	2009	PRJNA244927	SAMN02851047
B8838	VGIIc	Washington	Human	2010	PRJNA244927	SAMN02851052
B8843	VGIIc	Oregon	Human	2010	PRJNA244927	SAMN02851053
B11352	VGIIc	Oregon	Human	2015	PRJNA388113	SAMN07738532
B11442	VGIIc	Oregon	Elk	2016	PRJNA388113	SAMN07738533
B11468	VGIIc	Oregon	Human	2016	PRJNA388113	SAMN07738534
B11473	VGIIc	Oregon	Dog	2016	PRJNA388113	SAMN07738535
B11592	VGIIc	Oregon	Cat	2016	PRJNA388113	SAMN07738536
B8825	VGIIc	Oregon	Human	2010	PRJNA244927	SAMN02851050
B9933	VGIIc	Oregon	Human	2012	PRJNA244927	SAMN02851055
HL B3	VGIIc	Oregon	Human	2007	PRJNA244927	SAMN02851057
HL B4	VGIIc	Oregon	Human	2008	PRJNA244927	SAMN02851058
B11441	VGII	Oregon	Elk	2016	PRJNA388113	SAMN07738537
B11591	VGII	Oregon	Dog	2016	PRJNA388113	SAMN07738538
B11766	VGII	N. Carolina^d^	Human	2016	PRJNA388113	SAMN07738539
IAL3225	VGII	Brazil	Human	1994	PRJNA244927	SAMN02851062
IAL3234	VGII	Brazil	Human	1998	PRJNA244927	SAMN02851063
IAL3243	VGII	Brazil	Human	2000	PRJNA244927	SAMN02851064
B8973	VGII	Hawaii	Human	2010	PRJNA244927	SAMN02851060
B9764	VGII	Washington	Cat	2012	PRJNA244927	SAMN02851061
B9816	VGII	Oregon	Cat	2012	PRJNA244927	SAMN02851054
WM 04.78	VGII	Colombia	Human	1998	PRJNA244927	SAMN02851077
WM 04.84	VGII	Brazil	Human	1986	PRJNA244927	SAMN02851078
WM 05.274	VGII	Colombia	Human	2002	PRJNA244927	SAMN02851079
WM 05.275	VGII	Colombia	Human	2001	PRJNA244927	SAMN02851080
WM 05.339	VGII	Colombia	Human	2005	PRJNA244927	SAMN02851081
WM 05.342	VGII	Colombia	Human	2005	PRJNA244927	SAMN02851082
WM 05.419	VGII	Brazil	Human	1988	PRJNA244927	SAMN02851083
WM 05.452	VGII	Brazil	Human	1995	PRJNA244927	SAMN02851084
WM 05.456	VGII	Brazil	Environmental	1994	PRJNA244927	SAMN02851085
WM 05.457	VGII	Brazil	Human	1995	PRJNA244927	SAMN02851086
WM 05.461	VGII	Brazil	Human	1997	PRJNA244927	SAMN02851087
WM 05.462	VGII	Brazil	Human	1997	PRJNA244927	SAMN02851088
WM 05.525	VGII	Brazil	Human	1997	PRJNA244927	SAMN02851089
WM 05.527	VGII	Brazil	Human	1997	PRJNA244927	SAMN02851090
WM 05.528	VGII	Brazil	Human	2001	PRJNA244927	SAMN02851091
WM 05.529	VGII	Brazil	Human	1997	PRJNA244927	SAMN02851092
WM 05.530	VGII	Brazil	Human	1999	PRJNA244927	SAMN02851093
WM 05.533	VGII	Brazil	Human	1997	PRJNA244927	SAMN02851094
WM 05.536	VGII	Brazil	Human	1997	PRJNA244927	SAMN02851095
WM 05.545	VGII	Brazil	Human	2001	PRJNA244927	SAMN02851096
WM 05.546	VGII	Brazil	Human	2001	PRJNA244927	SAMN02851097
WM 05.547	VGII	Brazil	Human	2001	PRJNA244927	SAMN02851098
WM 05.76	VGII	Greece	Human	1996	PRJNA388113	SAMN07738540
WM 05.77	VGII	Greece	Human	1998	PRJNA388113	SAMN07738541
WM 06.12	VGII	Venezuela	Human	1996	PRJNA244927	SAMN02851099
WM 06.33	VGII	Aruba	NA	1953	PRJNA388113	SAMN07738542
WM 06.8	VGII	Uruguay	Environmental	1996	PRJNA388113	SAMN07738543
WM 08.309	VGII	Australia	Veterinary	1997	PRJNA244927	SAMN02851100
WM 08.311	VGII	Australia	Veterinary	1996	PRJNA244927	SAMN02851101
WM 09.152	VGII	Australia	Environmental	2009	PRJNA244927	SAMN02851103
WM 09.83	VGII	Australia	Human	1985	PRJNA388113	SAMN07738544
WM 09.94	VGII	Australia	Veterinary	2001	PRJNA244927	SAMN02851102
WM 11.65	VGII	Australia	Veterinary	2011	PRJNA244927	SAMN02851104
WM 178	VGII	Australia	Human	1991	PRJNA244927	SAMN02851073
WM 1850	VGII	Venezuela	Human	1999	PRJNA244927	SAMN02851074
WM 1851	VGII	Venezuela	Human	1999	PRJNA244927	SAMN02851075
WM 3032	VGII	Australia	Human	1983	PRJNA244927	SAMN02851076
HL A2	VGII	Brazil	Human	Pre-2008	PRJNA244927	SAMN02851065
HL A4	VGII	Uruguay	Environmental	1996	PRJNA244927	SAMN02851066
HL A5	VGII	Brazil	Environmental	Pre-2008	PRJNA244927	SAMN02851067
HL A6	VGII	Aruba	Veterinary	1953	PRJNA244927	SAMN02851068
HL A8	VGII	Brazil	Environmental	Pre-2008	PRJNA244927	SAMN02851069
HL B11	VGII	Greece	Human	1996	PRJNA244927	SAMN02851071
HL B12	VGII	Greece	Human	1998	PRJNA244927	SAMN02851072
HL B2	VGII	Brazil	Veterinary	2000	PRJNA244927	SAMN02851070

a The 22 samples newly sequenced for this study have BioProject accession number PRJNA388113.

b NA, not available (year of collection or sample source is unknown).

c NS, Canada, Nova Scotia, Canada.

d N. Carolina, North Carolina.

Genomic DNA was extracted from the 22 isolates using the ZR fungal/bacterial DNA MiniPrep kit (Zymo Research), following the manufacturer’s instructions. DNA samples were fragmented by sonication and prepared for multiplexed, paired-end sequencing with a 700-bp insert using the library hyper-preparation kit with standard PCR library amplification (KAPA Biosystems) as previously described (32). Libraries were quantified using the KAPA library quantification kit (KAPA Biosystems) and sequenced to a read length of 300 bp on the MiSeq instrument (Illumina, Inc., San Diego, CA).

### SNP matrix generation.

Sequenced genomes were assembled *de novo* using SPAdes version 3.6.0 (50). Reference genomes for phylogenetic analyses were selected based on the assembly quality metric *N*_50_ and total assembly length (see Table S1 in the supplemental material). NASP (51) was used to generate an SNP matrix for each of the three *C. gattii* subtypes, as well as the VGII complex, using the selected reference genomes for each genotype and R265 for the VGII lineage analysis. In brief, sequencing reads were aligned to the reference genome using Novoalign (Novocraft Technologies Sdn Bhd) and SNPs were identified using the GATK Unified Genotyper ToolKit version 2.7-2 (52). NASP filtered SNP loci not present in every sample and with less than 10× coverage and less than a 90% consensus in any sample. Using NUCmer (53), duplicated regions identified within the reference genome were also removed.

10.1128/mSphere.00499-17.7 TABLE S1  Reference genome assembly statistics, including number of contigs, number of contigs larger than 500 bases, *N*_50_, largest contig, and total size. Genomes were assembled using SPAdes. Download TABLE S1, XLSX file, 0.03 MB. Copyright © 2018 Roe et al. 2018 Roe et al. This content is distributed under the terms of the Creative Commons Attribution 4.0 International license.

### Phylogenetic analyses and root-to-tip regression.

IQ-Tree version 1.3.10 (54) was used to infer the best-fitting nucleotide substitution model for each of the three SNP matrices and to produce maximum-likelihood trees with 1,000 nonparametric bootstrap pseudoreplicates for branch confidence. Trees were visualized in FigTree version 1.4.2, and SNP numbers placed on branches using phangorn (55). To test each SNP matrix for evidence of recombination, which can confound divergence-dating analyses (18), the PHI test was conducted using PHIPack (56). In order to assess the temporal signal of the data set, regression analysis implementing root-to-tip genetic distance as a function of the sample collection year was conducted using the software package TempEst version 1.5 (http://tree.bio.ed.ac.uk/software/tempest/). Using the determination coefficient, *R*^2^, a measure of clocklike behavior was assessed. In an effort to maximize *R*^2^, the best-fitting root was selected based on TempEst recommendations (18). Additionally, we performed 10,000 random permutations of the sampling dates over the sequences in an effort to evaluate the significance of our regression results (57).

### Divergence time analyses.

A Bayesian molecular clock using tip dating was implemented in the BEAST version 1.8.0 software package (58) to infer evolutionary rates and time to most recent common ancestor (TMRCA) for the three VGII genotypes. MEGA7 (14) was used for nucleotide substitution model selection for each genotype, utilizing the Bayesian information criterion results to determine the best-fitting models. As previously described (32), available nucleotide substitution models are limited in BEAST, and therefore, the best-fitting model from MEGA7 that was also available in the BEAST software was implemented. While the SNP matrices only included variable sites, we corrected for the invariant sites by specifying a Constant Patterns model in the BEAST XML file. For each separate analysis, we determined the numbers of constant A’s, C’s, T’s, and G’s and added them to the XML file. Additionally, “path and stepping stone” sampling marginal-likelihood estimators were used in order to determine the best-fitting clock and demographic model combinations (59). The statistical fits of 10 different clock and demographic model combinations were assessed using the log marginal likelihood (Table S2). In BEAST, four independent chains of 4 billion iterations each were run for all molecular clock and demographic model combinations for the VGIIa and VGIIc data sets. Convergence among the four chains for VGIIb analysis completed after 1 billion iterations.

10.1128/mSphere.00499-17.8 TABLE S2  Metrics from BEAST analysis of VGIIa, VGIIb, and VGIIc SNP matrices. Metrics are provided for both relaxed lognormal clock and relaxed clock. Download TABLE S2, XLSX file, 0.04 MB. Copyright © 2018 Roe et al. 2018 Roe et al. This content is distributed under the terms of the Creative Commons Attribution 4.0 International license.

### Accession number(s).

All new sequence data files were deposited in the NCBI Sequence Read Archive (BioProject accession number PRJNA388113).
