# Supplementary figures and images for: Dating the Cryptococcus gattii Dispersal to the North American Pacific Northwest
Source: mSphere. 2018 Jan 17;3(1):e00499-17. doi: 10.1128/mSphere.00499-17 (PMC5770541; doi:10.1128/mSphere.00499-17)

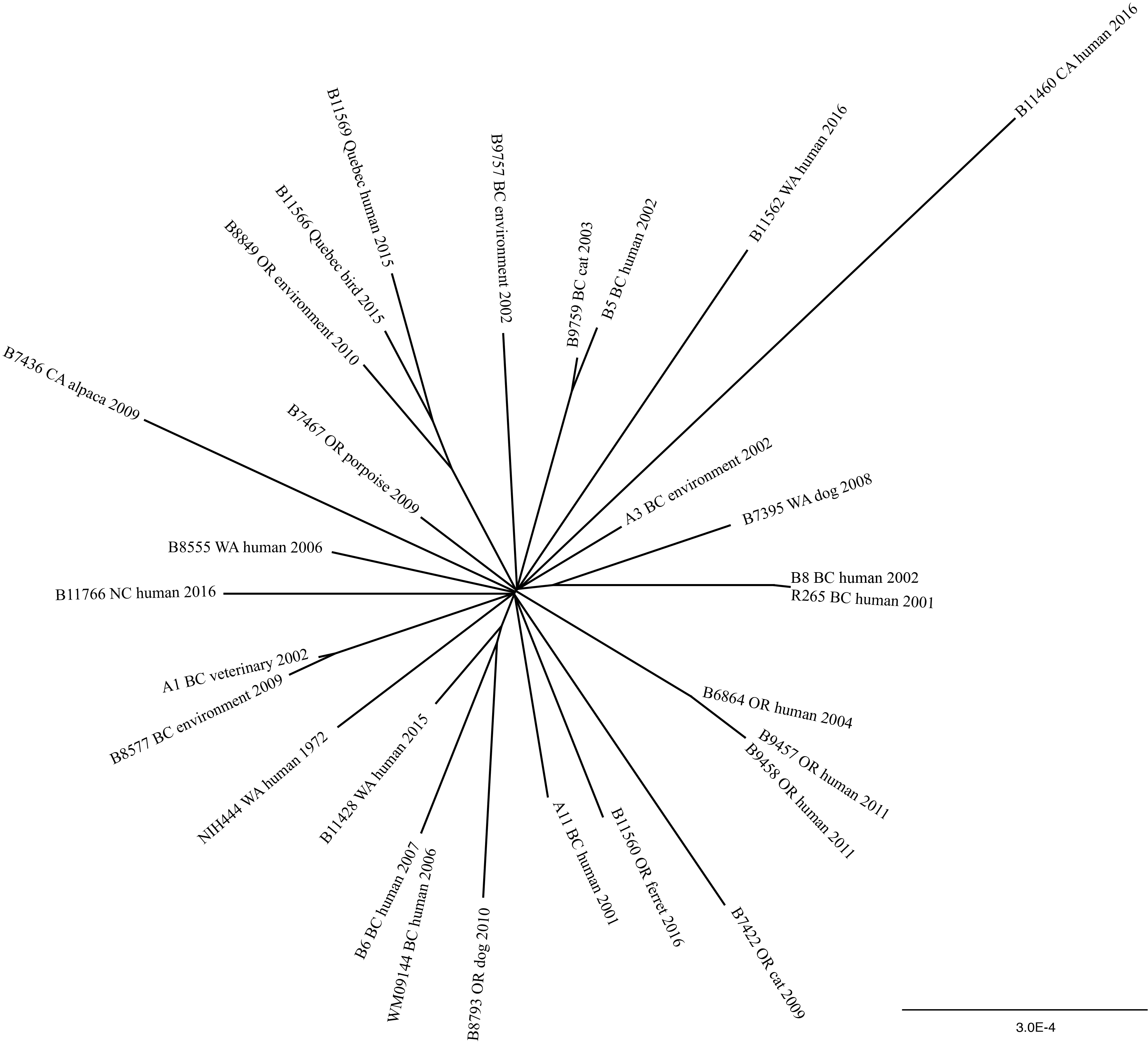

Supplement: FIG S1 [file sph001182456sf1.jpg]

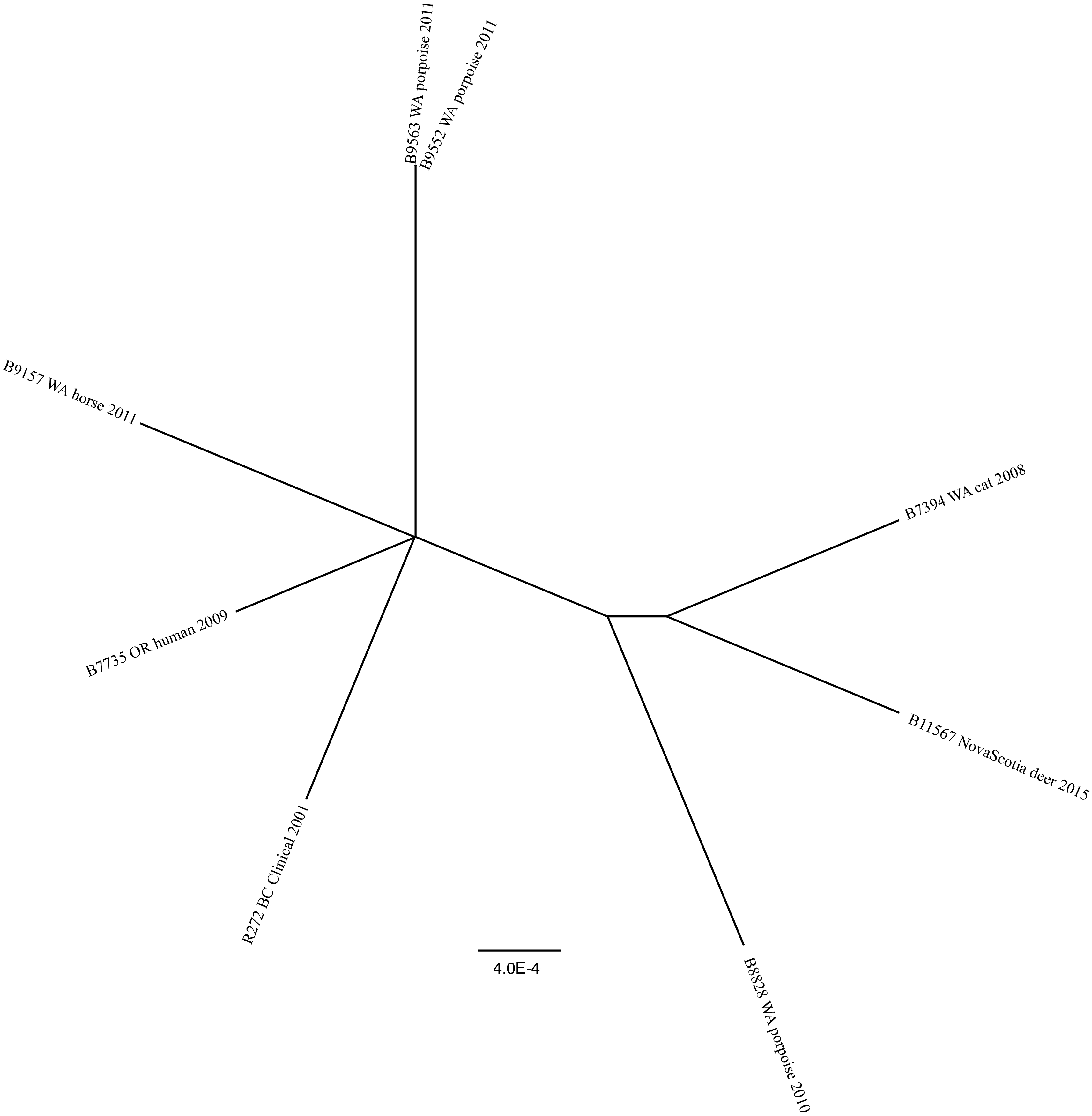

Supplement: FIG S2 [file sph001182456sf2.jpg]

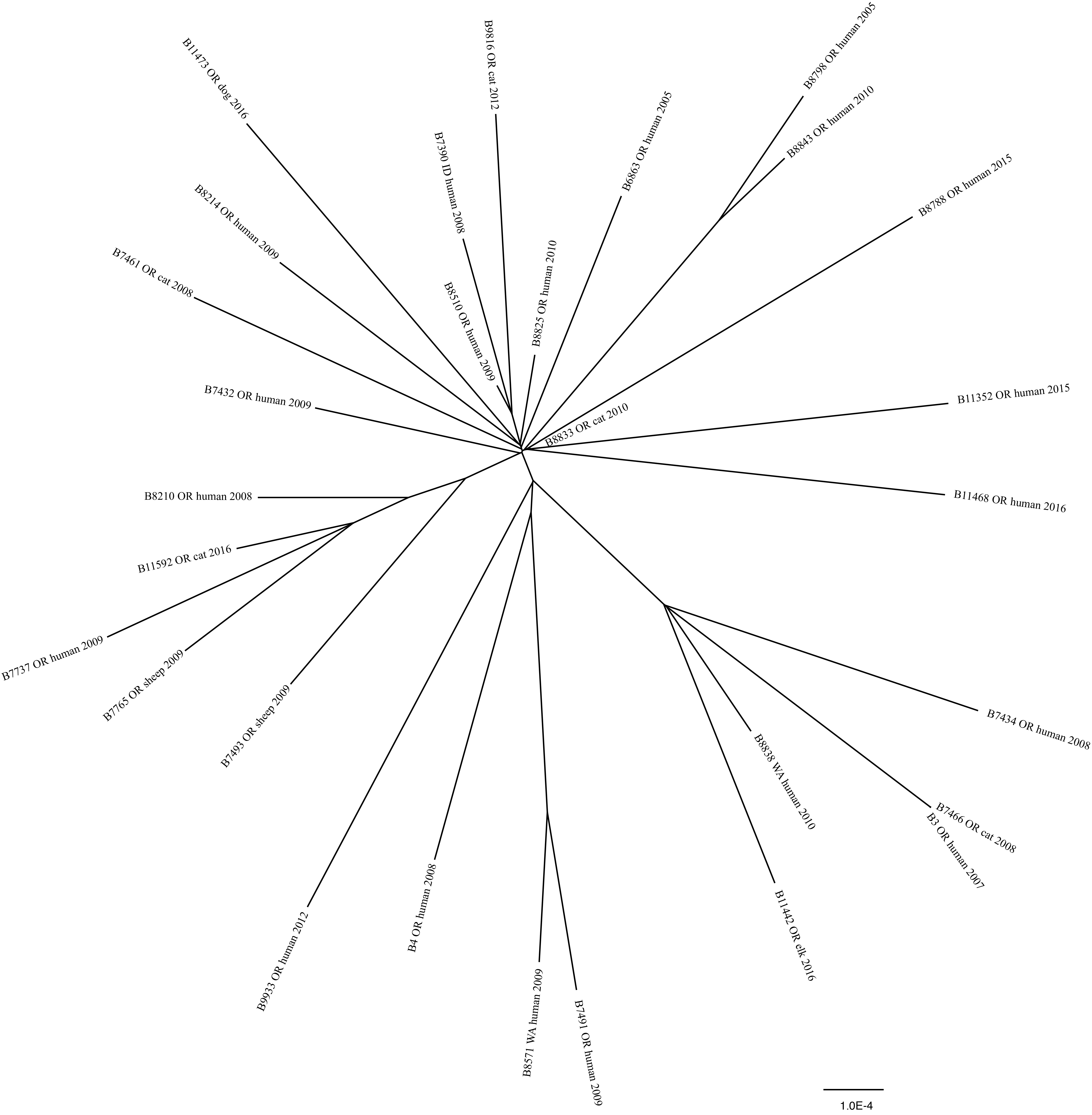

Supplement: FIG S3 [file sph001182456sf3.jpg]

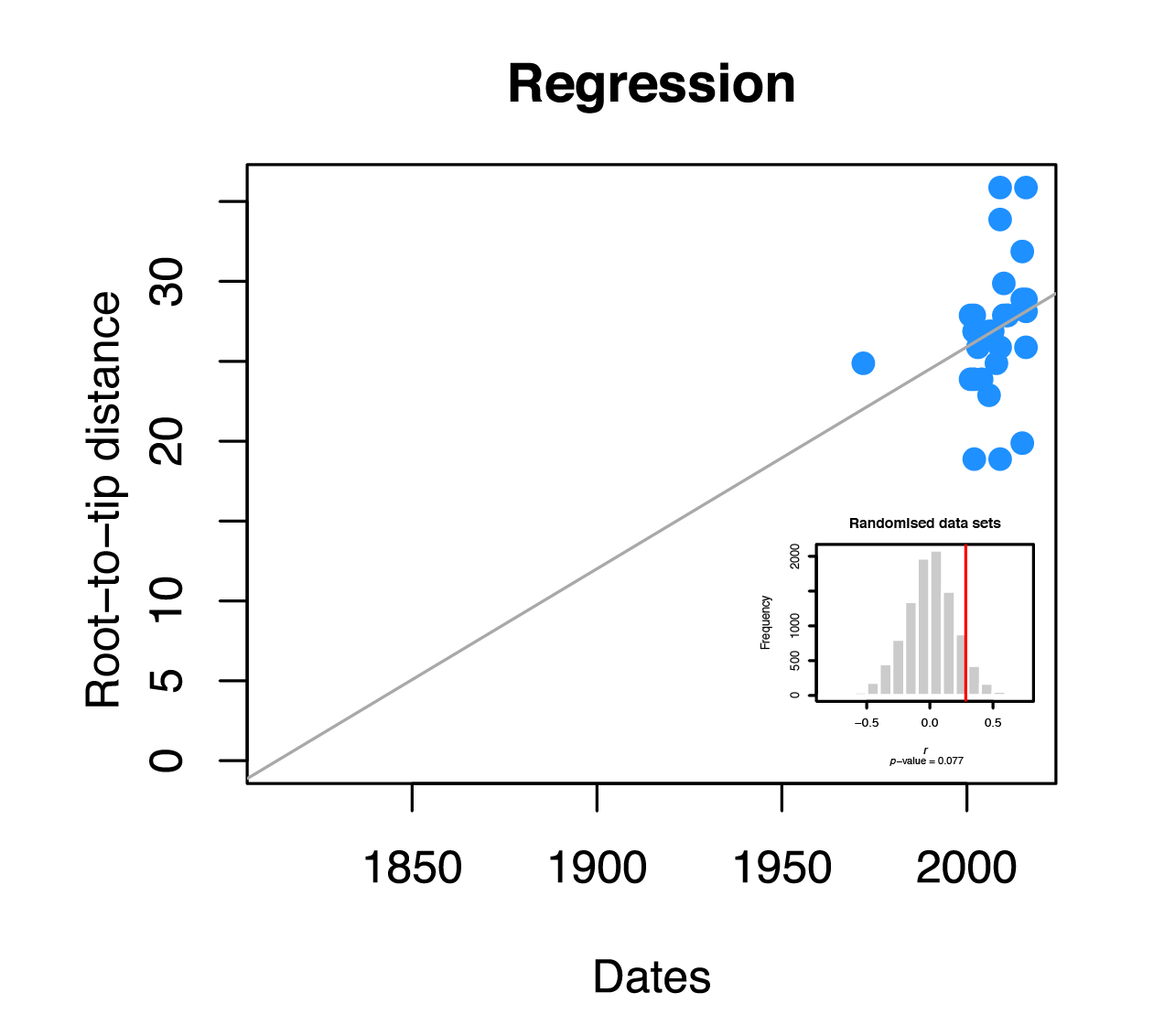

Supplement: FIG S4 [file sph001182456sf4.jpg]

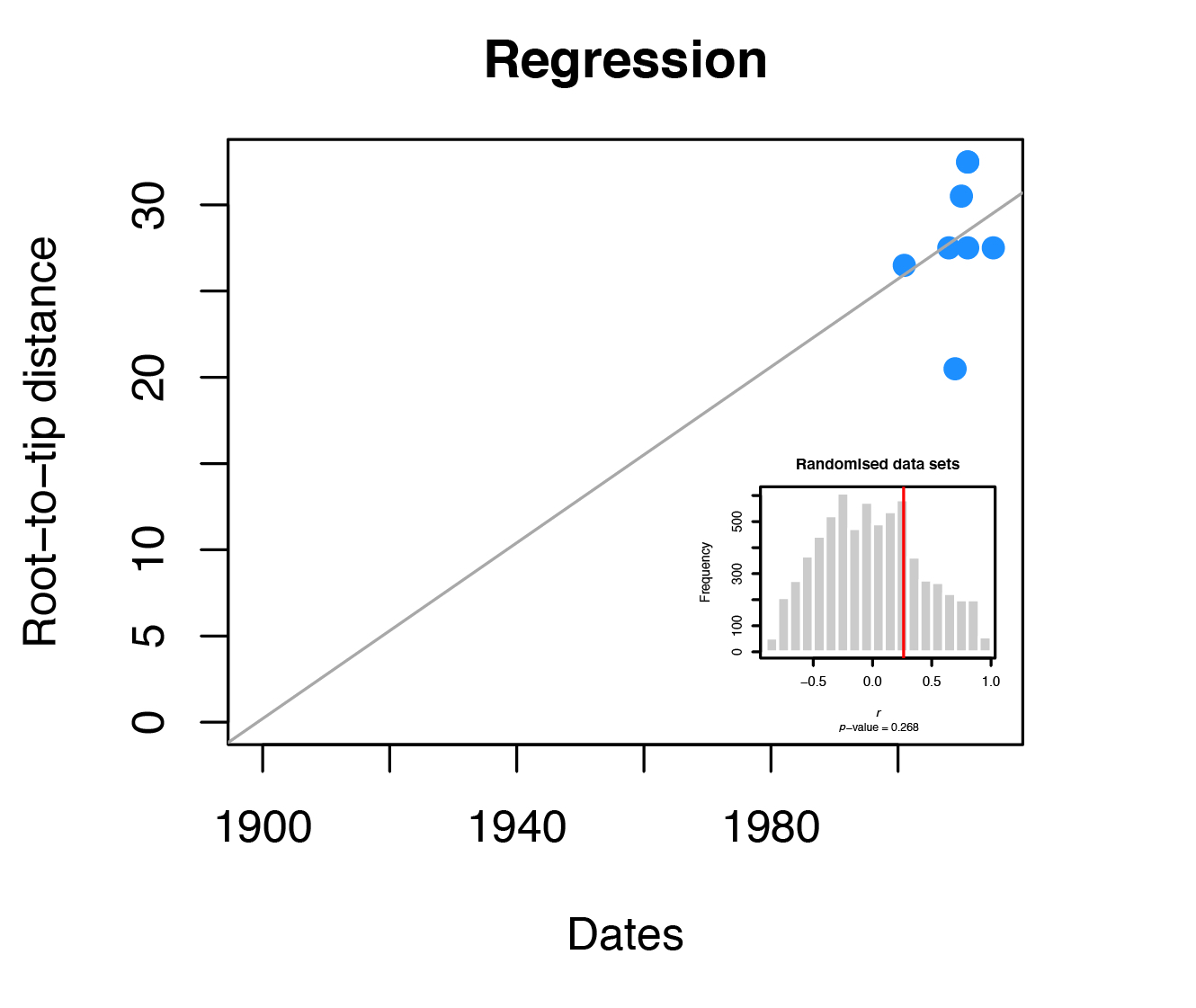

Supplement: FIG S5 [file sph001182456sf5.jpg]

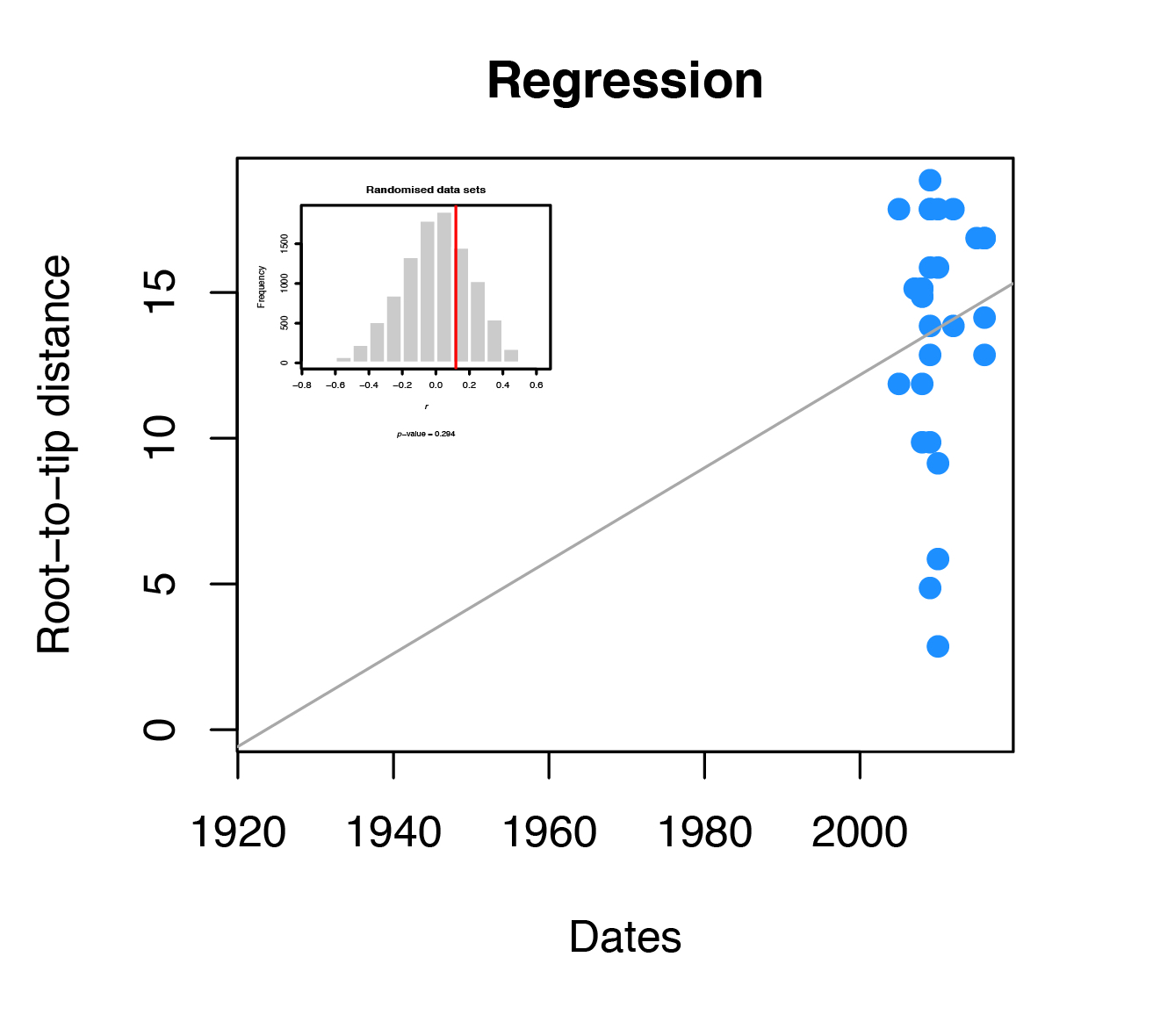

Supplement: FIG S6 [file sph001182456sf6.jpg]
